# HSDSnake: a user-friendly SnakeMake pipeline for analysis of duplicate genes in eukaryotic genomes

**DOI:** 10.1093/bioinformatics/btaf325

**Published:** 2025-05-28

**Authors:** Xi Zhang, Yining Hu, David Roy Smith, Zhenyu Cheng, John M Archibald

**Affiliations:** Department of Biochemistry and Molecular Biology, Dalhousie University, Halifax, NS B3H 4R2, Canada; Institute for Comparative Genomics, Dalhousie University, Halifax, NS B3H 4R2, Canada; Department of Computer Science, Western University, London, ON N6A 5B7, Canada; Department of Biology, Western University, London, ON N6A 5B7, Canada; Institute for Comparative Genomics, Dalhousie University, Halifax, NS B3H 4R2, Canada; Department of Microbiology and Immunology, Dalhousie University, Halifax, NS B3H 4R2, Canada; Department of Biochemistry and Molecular Biology, Dalhousie University, Halifax, NS B3H 4R2, Canada; Institute for Comparative Genomics, Dalhousie University, Halifax, NS B3H 4R2, Canada

## Abstract

**Summary:**

Gene duplication is a well-known driver of molecular evolution—it acts as a source of genetic novelty, thereby providing the raw substrate for organismal adaption. However, detecting different types of gene duplicates and comparing them in sequence datasets can be difficult. Existing tools can identify and classify gene duplicates that have arisen by various processes, but have limitations; for example, some do not have a user-friendly workflow and can include many intermediate steps requiring manual adjustments of parameters and/or are not maintained for the benefit of research community members. Here, we have developed HSDSnake, a user-friendly SnakeMake pipeline that can detect and classify gene duplications into five categories: dispersed, proximal, tandem, transposed, and whole genome. It also curates and evaluates the highly similar gene duplicates (HSDs) in each gene duplication category with reliance on both sequence similarity and conserved domains. Lastly, the detected gene duplicates can be visualized within a KEGG functional pathway framework and the substitution rates (*Ka*, *Ks*, and their *Ka/Ks* ratio) can be analyzed for all the duplicate gene pairs. We demonstrate HSDSnake’s capabilities by analyzing two reference genomes directly downloaded from NCBI and provide detailed instructions for each step.

**Availability and implementation:**

The HSDSnake pipeline uses SnakeMake and Conda to run and install dependencies. The distribution version is available online at GitHub: https://github.com/zx0223winner/HSDSnake and the archived version at Zenodo is https://doi.org/10.5281/zenodo.15521945.

## 1 Introduction

The origin and evolution of duplicate genes is a topic that has long fascinated molecular biologists. With the explosion in the amount of genome data available in public databases such as the National Center for Biotechnology Information (NCBI) and other online resources (Pruitt *et al.* 2005, Schoch *et al.* 2020), it is possible to study the phenomenon of gene duplication in great detail and in a wide range of species. Competing hypotheses for the origin and retention of duplicate genes have been proposed (Innan and Kondrashov 2010). For example, Ohno’s neofunctionalization model (Ohno 1970), the “Escape from adaptive conflict” model (Des Marais and Rausher 2008) and the gene dosage hypothesis (Qian and Zhang 2008) explore how duplicate genes can be fixed and maintained through adaptive evolution. Conversely, the appearance and loss of duplicate genes from genomes can also be interpreted using neutral theories, which argue that genetic drift might be the primary drivers of duplicate gene evolution (Nei and Roychoudhury 1973, Li 1980, Lynch 2007, Brunet and Doolittle 2018). Research suggests that the likelihood of retention of sensory, transport, and stress response genes is impacted by environmental conditions (Kondrashov 2012). Hundreds of highly similar duplicate genes (HSDs) were recently identified in the genome of the Antarctic green alga *Chlamydomonas priscuii* and the snow alga *Sanguina aurantia*, organisms in which gene dosage appears to aid in their survival (Cvetkovska *et al.* 2018, Zhang *et al.* 2021a, Stahl-Rommel *et al.* 2022, Raymond *et al.* 2024).

Given the abundance and complexity of duplicate genes in nuclear genome sequence data, it is increasingly popular to apply automated prediction and analysis pipelines to speed up research and reduce labor-intensiveness. Tools and software have been developed for identifying duplications within and between genomes at various scopes and scales (Lallemand *et al.* 2020, Zhang and Smith 2022), taking advantage of a rule-based approach to workflow establishment (Conery *et al.* 2005). For example, GenomeHistory (Conant and Wagner 2002) was developed over twenty years ago and can identify all pairs of duplicate genes and calculate the synonymous and non‐synonymous substitutions per nucleotide site (*Ka* and *Ks*) between duplicate pairs. More recently, integrated tools such as MCScanX (Wang *et al.* 2012, Wang *et al.* 2013, Wang *et al.* 2024), i-ADHoRe (Proost *et al.* 2012) and CYNTENATOR (Rödelsperger and Dieterich 2010) were developed to search for syntenic blocks (mainly for detecting whole genome and segmental duplications) (Liu *et al.* 2018). OrthoDB (Zdobnov *et al.* 2017) and OrthoMCL (Li *et al.* 2003) use a graph-based method and Markovian Cluster algorithm to identify in-paralogs (i.e. duplicate genes) within species. Likewise, OrthoFinder (Emms and Kelly 2015, 2019) can detect orthogroups across species and infer gene duplication events from phylogenetic trees. For tools that detect duplicate genes based on paralogous relationships, sequence similarity is usually measured by percent identity, aligned length difference, and E-value (Lallemand *et al.* 2020). Alignment tools such as BLAST (Kent 2002), DIAMOND (Buchfink *et al.* 2015) and nhmmer (Wheeler and Eddy 2013) can generate these metrics, but this can come with a steep learning curve associated with managing installation dependencies under different computational working environments. Many recently developed bioinformatic tools are designed to prioritize analytical flexibility over user-friendliness; they can include many parameters that present challenges for researchers lacking computational experience. For example, the DupGen_finder pipeline (Qiao *et al.* 2019) can detect and classify duplicates into five duplication categories [dispersed (DD), proximal (PD), tandem (TD), transposed (TRD), and whole genome (WGD)] using a built-in MCScanX algorithm (Wang *et al.* 2012, 2013), but the pipeline was written in Perl and lacks a standard data input format. This means it is not straightforward to reproduce users’ own data. More recently, a MCScanX protocol (Wang *et al.* 2024) was released with detailed steps for implementing synteny and evolutionary analyses; however, it is difficult to perform quality control on a step-by-step basis, and is challenging to set up different environment dependencies for new users.

To aid in the task of analyzing duplicate genes in eukaryotic genomes, we have developed HSDSnake, a SnakeMake-based user-friendly solution that not only integrates tools developed previously, namely the HSDFinder web tool (Zhang *et al.* 2021b, 2021c), the HSDatabase online platform (Zhang *et al.* 2022), and HSDecipher (Zhang *et al.* 2023), but also integrates scripts from other recently developed methods and tools, such as DupGen_finder (Qiao *et al.* 2019) and MCScanX (Wang *et al.* 2024). HSDSnake thus represents a substantial improvement over our previously developed tools; it not only classifies gene duplicates into the above-mentioned duplication types (i.e. DD, PD, TD, TRD, and WGD), but can also calculate substitution rates between duplicate pairs (*Ka*, *Ks*, and *Ka/Ks* ratio). Furthermore, the custom scripts in HSDSnake smoothly move users from one analysis step to the next, allowing comprehensive detection, curation, visualization, and comparison of gene duplicates contained within and between genomes. It can efficiently analyze duplicated genes in unannotated sequences in a single or multi-genome format by integrating the results from InterProScan (Quevillon *et al.* 2005, Mitchell *et al.* 2019) and KEGG databases (Kanehisa and Goto 2000). The results of the predicted HSDs can be further curated by tweaking thresholds, evaluated by various performance metrics, and then visualized in publication-ready heatmaps. At present there are very few tools that can detect highly similar duplicate genes (HSDs) with reliance on both sequence similarity and conserved domains in intra-species genomes and execute downstream comparative genomics analysis. Here, two reference genomes were used as a study case to demonstrate HSDSnake’s capabilities.

## 2 Implementation

HSDSnake combines a set of custom Python and Perl scripts with built-in DupGen finder, HSDecipher and HSDFinder tools to support synthetic analysis of HSDs in eukaryotic genomic data. The software implementation follows a standard SnakeMake pipeline structure with default config and snakemake files. The Conda environment files (mcscanx.yaml, diamond.yaml, hsdfinder.yaml, hsdecipher.yaml, etc.) reside in the environment folder. The customs scripts are written in Python 3 and Unix command lines, which have been automatically integrated into the pipeline. The scripts folder contains the cores scripts from the DupGen finder, MCScanX_protocol, HSDecipher, and HSDFinder tools. Figure S1, available as supplementary data at *Bioinformatics* online, summarizes the HSDSnake workflow, which includes three parts. In the first two parts of the pipeline, the file preparation section requires a config file (config.yaml) to access the necessary input files, such as that used for standard format genomic data downloaded directly from NCBI (“XX.genomic.gff,”, “XX.protein.faa,” and “XX.cds_from_genomic.fna”). In the second part, HSDSnake can integrate scripts from DupGen_finder (Qiao *et al.* 2019) and MCScanX (Wang *et al.* 2024) to detect and classify gene duplicates into five duplication types (DD, PD, TD, TRD, and WGD). The third part of the pipeline can perform the calculation and visualization of substitution rates per substitution site for the detected gene pairs (*Ka*, *Ks*, and *Ka/Ks* ratio).

HSDSnake can curate and evaluate HSDs in each detected gene duplication category with reliance on both sequence similarity and conserved domains. This portion of the workflow is divided into three sections and seven steps. In steps 1–3, HSDSnake requires the following external necessary input files: (i) the InterProScan (Quevillon *et al.* 2005, Mitchell *et al.* 2019) search results using protein sequences as queries, and (ii) gene lists with KO annotations from the KEGG database (Kanehisa and Goto 2000). Since amino acid substitutions occur less frequently than nucleotide substitutions, protein sequence alignments are more informative than nucleotide alignments for many comparative genomic applications (Koonin and Galperin 2003). Distinct from our previous HSD detecting tools, HSDSnake utilizes Diamond blastp all-against-all searches (Buchfink *et al.* 2015) (defaulted parameters: E-value cut-off ≤ 1e-10, blastp -outfmt 6 etc.). In steps 4–6, the built-in custom scripts of HSDSnake pre-process the input fasta sequence data into an appropriate format and feed the input data files (e.g. species_name.dup_type.preprocess.txt, species_name.interproscan.tsv and species_name.ko.txt) into the built-in HSDFiner tool (Zhang *et al.* 2021b). HSDFinder categorizes gene duplicates by considering user-provided thresholds (i.e. pairwise amino acid identity and variances) and annotates the duplicates based on protein functional domains and pathway information from the Pfam and KEGG databases. Since there is no simple rule for distinguishing partial duplicates from more complete ones, another built-in tool, HSDecipher (Zhang *et al.* 2023), is used to curate the HSD results. A series of similarity assessment metrics is employed to strategically expand the dataset of HSDs (e.g. from 50% to 100% pairwise amino acid identity and between 10 to 100 amino acid differences). In this way, HSD datasets generated using certain thresholds can be automatically enlarged and curated simply by changing the thresholds. For example, HSDs identified at a threshold of 70%_50aa can be added to those identified at a threshold of 70%_30aa (denoted as “70%_50aa + 70%_30aa”). If the more relaxed threshold (i.e. 70%_50aa) contains identical genes acquired using the stricter cut-off (70%_30aa), the combined HSD candidate list can be filtered to remove the redundancy.

In Step 8, the built-in scripts of HSDecipher generate HSD statistics and categories to help users evaluate the composition and utility of the HSD dataset, thereby helping to best align it with the research question at hand. In the third section, the HSDSnake workflow facilitates visualization of intra-/inter-genomic HSD data using a heatmap, which shows the functional distribution of HSDs or the levels of HSD sequence similarity shared between different species or between duplicates within a single genome. Significantly enriched HSDs can be easily visualized and compared, alongside a tabular file for comparing HSDs with the same KEGG pathway function. This allows users to conveniently choose HSDs of particular interest for additional analysis (e.g. identification of signatures of natural selection).

## 3 Application

The HSDSnake pipeline output constitutes a comprehensive analytical framework with method descriptions, tables, and heatmap visualizations, enabling detailed interrogation of duplicate gene data in genomic datasets. To run locally, pre-installed Python (preferably Python 3) and Linux (e.g. Ubuntu 20.04 LTS) environments are suggested. To demonstrate the abilities and outputs of the pipeline, two reference genomes (the land plant *Arabidopsis thaliana* and the green alga *Chlamydomonas reinhardtii*) were downloaded from NCBI (e.g. GCF_000001735.4.zip and GCF_000002595.2.zip) and analyzed; these two genomes have 48,265 and 19,527 predicted protein-coding genes, respectively. In the first and second parts of the pipeline, each diamond blast and *Ka/Ks* calculation took around 8 min for each species. For the third part, HSDs detection, curation, and visualization took less than 10 min for each species.

As shown in Text S1 and S2, available as supplementary data at *Bioinformatics* online, we first checked if there are multiple isoforms derived from alternative splicing and choose the one with longest transcript length as the primary protein sequence. Then we applied the pre-processing scripts from the MCScanX protocol (Wang *et al.* 2024) to generate the genome annotation file (e.g. Athaliana.gff) and the coding sequence file (Athaliana.cds). The reciprocal blast all-and-all results (e.g. Athaliana.blast) were then generated using Diamond blastp with default parameters (Buchfink *et al.* 2015) (e.g. E-value cut-off ≤ 1e-10, blastp -outfmt 6 etc.). Lastly, *C. reinhardtii* was used as an outgroup species which is helpful for inter-genomic comparisons (e.g. Athaliana_Creinhardtii.blast), allowing detection of other types of duplicates in the *A. thaliana genome*.

With these input files in hand, we applied the core DupGen_finder pipeline scripts (DupGen_finder.pl and DupGen_finder-unique.pl) to detect and classify the gene duplicates into five categores (DD, PD, TD, TRD, and WGD) (Text S3, available as supplementary data at *Bioinformatics* online). Comparing the classifying results from an example used in the DupGen_finder pipeline (Qiao *et al.* 2019), the frequencies of duplicate pairs per mode were similar for most pairs, although there are some differences for transposed duplicates (TRDs: 4,447 were reported in DupGen_finder compared to 4,861 in HSDSnake). These differences may be due to the fact that while *A. thaliana* was used as a test case for both tools, the original DupGen_finder analysis used a different outgroup species (*Nelumbo nucifera*). A factor that can impact the PD detection is parameter preference (-d, default 10), which is the threshold for how many genes are considered as PD (790 PDs were reported in DupGen_finder compared to 870 detected here with HSDSnake). Since the DupGen_finder pipeline (Qiao *et al.* 2019) was designed to detect gene duplicates in sequence (one detected after another), dispersed duplicates (DDs) are assigned only when the other four duplication categories are excluded, which helps explain where the observed differences are coming from.

As also shown in Text S3, available as supplementary data at *Bioinformatics* online, HSDSnake is able to calculate and visualize *Ka*, *Ks* and *Ka/Ks* ratios for all the detected gene duplicates pairs using the scripts from the MCScanX protocol (Wang *et al.* 2024). The relevant table (e.g. Athaliana.collinearity.kaks) and figure (e.g. A thaliana.synteny.blocks.ks.distri.pdf) are presented in the tutorial. For example, a distribution curve of Ks values of syntenic blocks within *A. thaliana* are created by fitting the Gaussian mixture models (GMM).

As shown in Text S4, available as supplementary data at *Bioinformatics* online, by applying scripts from HSDFinder and HSDecipher tools, the duplicate gene pairs were curated to allow further interpretation. We applied the custom scripts *HSD_add_on.py* and *HSD_batch_run.py* to assemble a larger dataset of HSD candidates by using a combination of thresholds (e.g. Athaliana_tandem.batch_run.txt). Noted that gene duplicates can have very different similarity levels within and between genomes; large sets of HSD candidates should be treated with caution, in particular those encoding ribosomal proteins and histones (Zhang 2003, Zhang and Smith 2022).

We then used two custom scripts (i.e. *HSD_statistics.py* and *HSD_categories.py*) to evaluate the results. The first summarized the HSD results into a useful HSD statistic using multiple HSDFinder thresholds. For example, the output tabular file (Athaliana_tandem.stat.txt) can reveal many valuable patterns in the HSD data, such as number of gene copies, non-redundant gene copies, and the performance of the HSDs to be captured. The latter script can count the number of HSDs whose gene copies are formed into different number of categories (Athaliana_tandem.category.txt). This step is important for users wanting to evaluate the distribution size of HSD groups. When comparing the duplicated gene pairs detected in DupGen_finder, HSDs represented the group of gene pairs share both the sequence similarity and conserved domains. Using *A. thaliana* as an example, HSD groups can be categorized into two, three, or more paired gene duplicates (occupied 66%, 18%, and 16% of total HSDs, respectively). In the *A. thaliana* test dataset, HSDs for each duplication class were as follows: DD = 802, PD = 213, TD = 602, TRD =511, and WGD = 1,988). Lastly, we applied the *HSD_heatmap.py* to visualize the HSD results in high resolution heatmaps (e.g. Athaliana_tandem.output_heatmap.eps and HSD.output_heatmap.eps) under a framework of biochemical pathway function. KO accession numbers corresponding to each gene duplicate in the pathway are collected from the KEGG database (Kanehisa and Goto 2000). Depending on the HSD data, the color matrix of the heatmap represents the number of HSDs sharing the same KO with each other in intra- or inter-genomes.

## 4 Limitation and future plan

The HSDSnake pipeline is designed to run on standard genomic data taken from NCBI. Those who want to use other data sources, such as Phytozome (Goodstein *et al.* 2012), must make sure the input data match the provided examples. For users who are new to workflow management tools, such as SnakeMake, there is admittedly learning curve, but the time investment is worth the effort given the rich data output for downstream analysis of gene duplicates. Users should note that InterProScan (Quevillon *et al.* 2005, Mitchell *et al.* 2019) and KEGG (Kanehisa and Goto 2000) are the only third-party tools not integrated in the HSDSnake pipeline due to the lack of a Conda environment (at present the latest InterProScan Conda package of 5.59 is not compatible with SnakeMake). We also note that there are limitations to web-only access to KEGG-associated tools such as BlastKOALA (Kanehisa *et al.* 2016). In this case, users may have to learn and execute third-party tools and prepare their input data manually. All things considered, however, HSDSnake is straightforward to run by checking the respective ReadMe files and following the protocols described previously in Zhang *et al.* (2021c). In the future, HSDSnake will be upgraded to allow more input data sources and new bioinformatics tools to allow even more sophisticated analysis of gene duplicates.

## 5 Conclusions

HSDSnake is designed to support comprehensive analysis of duplicate genes in nuclear genomic data, with an emphasis on highly similar duplicates. It integrates the latest duplication detection tools and our previously developed HSD resources. The built-in custom SnakeMake file allows users to proceed from one analysis step to the next, producing detailed outputs with method descriptions, tables, and heatmap visualizations. With its user-friendly approach to duplicate gene analysis, HSDSnake thus fills a need for the bioinformatics and genomics community.

## Supplementary Material

btaf325_Supplementary_Data
