# Supplementary material for: HSDSnake: a user-friendly SnakeMake pipeline for analysis of duplicate genes in eukaryotic genomes
Source: Bioinformatics. 2025 May 28;41(6):btaf325. doi: 10.1093/bioinformatics/btaf325 (PMC12202878; doi:10.1093/bioinformatics/btaf325)
Supplement: btaf325_Supplementary_Data [file btaf325_supplementary_data.pdf]

Supplementary materials for HSDSnake pipeline

Xi Zhang[1,2*], Yining Hu[3], David Roy Smith[4], Zhenyu Cheng[2,5], John M. Archibald[1,2*]

[1]Department of Biochemistry and Molecular Biology, Dalhousie University, Halifax, Nova Scotia, B3H 4R2, Canada.

[2]Institute for Comparative Genomics, Dalhousie University, Halifax, Nova Scotia, B3H 4R2, Canada.

[3]Department of Computer Science, Western University, London, Ontario, N6A 5B7, Canada.

[4]Department of Biology, Western University, London, Ontario, N6A 5B7, Canada.

[5]Department of Microbiology and Immunology, Dalhousie University, Halifax, Nova Scotia, Canada.

*Correspondence: xi.zhang@dal.ca (X.Z.)

*Correspondence: john.archibald@dal.ca (J.M.A.)

*To whom correspondence should be addressed.

May 26th, 2025

Part 1-2 of the Pipeline

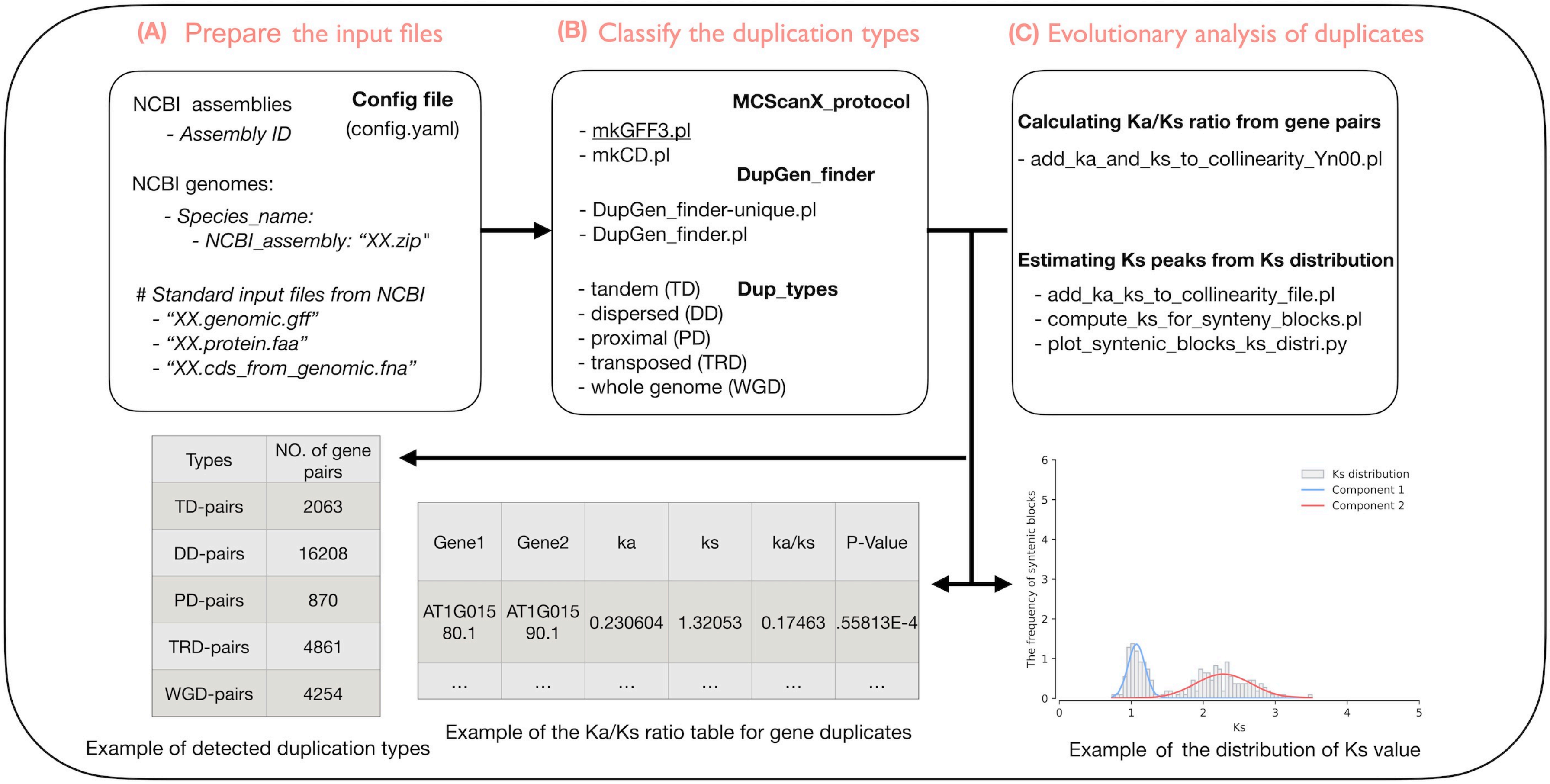

Part 3 of the Pipeline

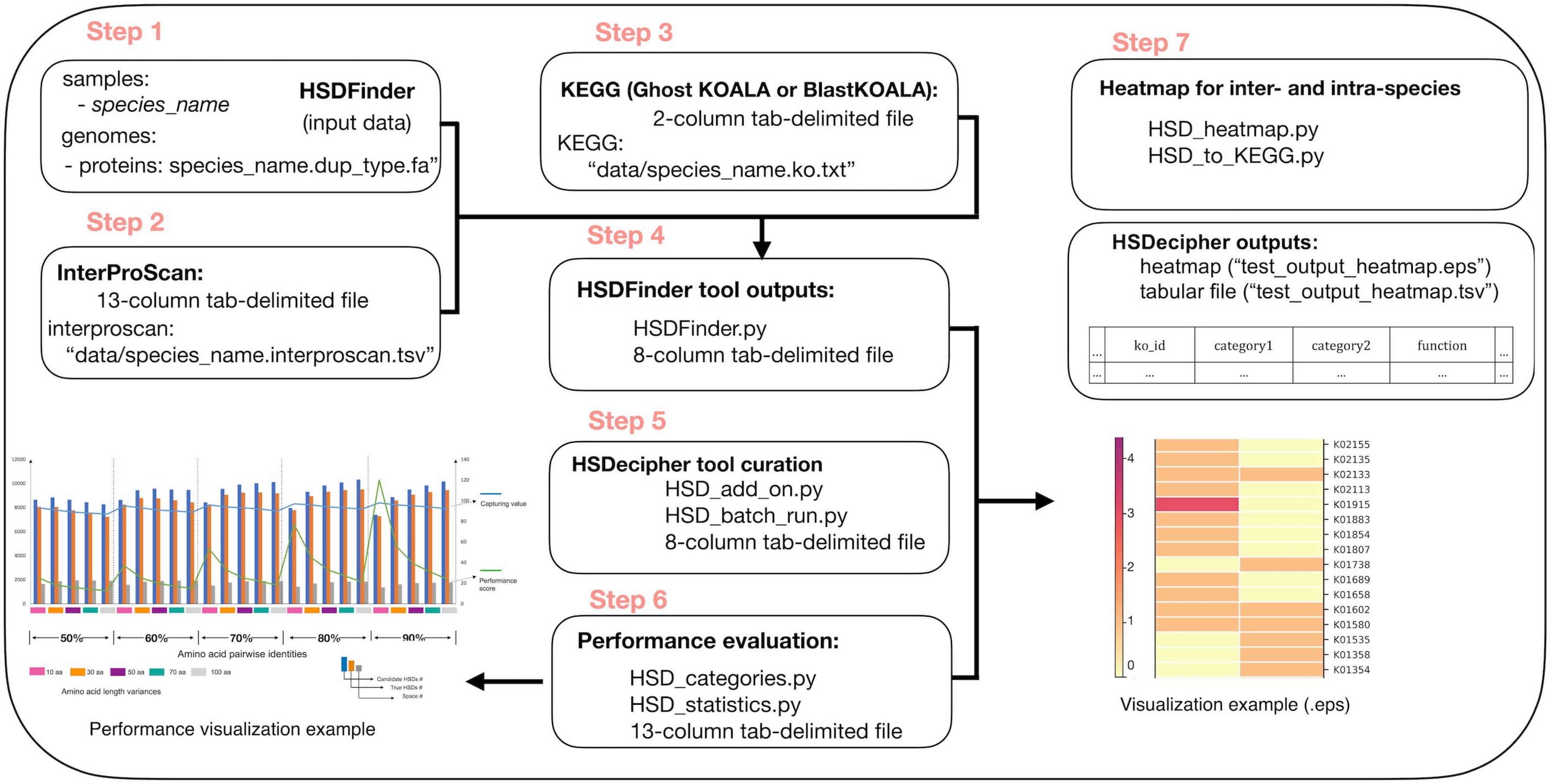

Supplementary Figure S1: HSDSnake workflow. Part 1-2: (A) Prepare the SnakeMake config file which contains the species name, out group name, genomic assembly ID and other input file directories. **(B)** Detect and classify the gene duplicate pairs into five different duplication types (DD, PD, TD, TRD, and WGD) via the scripts from the DupGen finder and MCScanX protocols. **(C)** Calculate and visualize the synonymous substitutions per site (Ks), non-synonymous substitutions per site (*Ka*), and their ratios (*Ka/Ks*) for each gene pair**; Part 3:** (1) Prepare the SnakeMake config file which contains different types of gene duplicates. (2) Prepare an InterProScan search result file of your genome in tab-separated values (tsv.). (3) Prepare a gene list with KO annotation from KEGG database. (4) Run the built-in HSDFinder tool and diamond BlastP all-against-all search; this will yield an HSD output file in tab-separated value (tsv.) format. (5) Curate the HSDs using the built-in HSDecipher downstream analysis tool with a combination of thresholds. (6) Evaluate the suitability of the results and visualize the performance outputs in a plot. (7) Visualize the curated HSD results from a single or multiple genome perspective in a heatmap and generate a detailed HSDs functional annotation tabular file. The plot of step 6 was adopted with permission (Zhang et al., 2021).

Supplementary Text: Usage of SnakeMake pipeline. Text S1. Introduction of the config.yaml file; Text S2. Download and preprocess the NCBI assemblies (Snakefile_part1); Text S3. Detect and classify gene duplication categories by DupGen_finder (Snakefile_part2); Text S4. Refine and visualize the gene duplicates with HSDFiner (Snakefile_part3).

📋Supplementary Text : Usage of SnakeMake pipeline.

Contents:

- Text S1. Introduction for the config.yaml file;
- Text S2. Download and preprocess the NCBI assemblies (Snakefile_part1);
- Text S3. Detect and classify gene duplication categories by DupGen_finder (Snakefile_part2);
- Text S4. Refine and visualize the gene duplicates with HSDFiner (Snakefile_part3).

Text S1. Config.yaml file

You will need to edit the config.yaml file for your own usage. An example config.yaml has been provided to test the pipeline.

> ⚠ Warning
>
> please only substitute the species name with yours, keep the input file format, such as Arabidopsis_thaliana.fa, Arabidopsis_thaliana.interproscan.tsv, Arabidopsis_thaliana.ko.txt

```
# Critical: input files for HSDSnake, please only substitute the specie
# such as Athaliana.interproscan.tsv, Athaliana.ko.txt

ncbi_assemblies:
  - GCF_000001735.4
  - GCF_000002595.2

ncbi_genomes:
    Athaliana:
        ncbi_assembly: "data/ncbi_download/GCF_000001735.4.zip"
        assembly_id: "GCF_000001735.4"
        outgroup: "Creinhardtii"
        interproscan: "data/Athaliana.interproscan.tsv"
        KEGG: "data/Athaliana.ko.txt"
        feature_table: "data/ncbi_download/GCF_000001735.4_TAIR10.1_fea
    Creinhardtii:
        ncbi_assembly: "data/ncbi_download/GCF_000002595.2.zip"
        assembly_id: "GCF_000002595.2"
        outgroup: "Athaliana"
        interproscan: "data/Creinhardtii.interproscan.tsv"
```

```
        KEGG: "data/Creinhardtii.ko.txt"
        feature_table: "data/ncbi_download/GCF_000002595.2_Chlamydomona

names:
  - Athaliana
  - Creinhardtii

dup_types:
  - tandem
  - dispersed
  - proximal
  - transposed
  - wgd
  - all

# Wang, Yupeng, et al. Nature Protocols 19.7 (2024): 2206-2229.
MCScanX_protocol:
 - "/scripts/MCScanX_protocol"
 - mkGFF3.pl
 - mkCD.pl
 - add_ka_and_ks_to_collinearity_Yn00.pl

# Qiao, Xin, et al. Genome biology 20 (2019): 1-23; Wang, Yupeng, et al
DupGen_finder:
 - "/scripts/DupGen_finder"
 - DupGen_finder-unique.pl
 - DupGen_finder.pl

# Qiao, Xin, et al. Genome biology 20 (2019): 1-23.
identify_Ks_peaks_by_fitting_GMM:
  - "/scripts/identify_Ks_peaks_by_fitting_GMM/"
  - add_ka_ks_to_collinearity_file.pl
  - compute_ks_for_synteny_blocks.pl
  - plot_syntenic_blocks_ks_distri.py

HSDFinder:
  - "/scripts/hsdfinder/"


################Above are the directories for fasta, interproscan, ko [illegible]

HSD_identity:
- 90
- 80
- 70
- 60
- 50

HSD_variance:
- 100
```

```
- 70
- 50
- 30
- 10

HSDecipher: "/scripts/hsdecipher/"

########Feel free to modidy the heatmap hight and width depending on yo

heatmap_hight: 20

heatmap_width: 30
```

Text S2. Snakefile_part1

Download NCBI assemblies

`Purpose`: This rule provides a convenient way to download the standard input files from NCBI.

> ⓘ Note
>
> To avoid repeatly download the ".zip" files with the example file we provided ('HSDSnake_data.tar.gz'), we commented the rule in the snakefile.

`scripts`:

```
mkdir -p {params.dir};\
curl -OJX \
GET "{params.link}"; \
mv {params.file} {params.dir} \
```

`Output`: data/ncbi_download/GCF_000001735.4.zip

```
# standard input files from NCBI
XX.genomic.gff
XX.protein.faa
XX.cds_from_genomic.fna
```

Preprocessing the naming of the NCBI assemblies

`Purpose` : Rename the NCBI genomic assembly to the format which mcscanx and Dupgen-finder can take.

`scripts` :

```
mkdir -p {params.dir2}{params.species_name}; \
unzip {params.dir1}{params.assembly_id}.zip -d {params.dir1}
{params.species_name}; \
sleep 5s; \
cp {params.dir1}
{params.species_name}/ncbi_dataset/data/{params.assembly_id}/cds_from_g
{params.dir2}
{params.species_name}/{params.species_name}_cds_from_genomic.fna; \
cp {params.dir1}
{params.species_name}/ncbi_dataset/data/{params.assembly_id}/genomic.gf
{params.dir2}
{params.species_name}/{params.species_name}_genomic.gff; \
cp {params.dir1}
{params.species_name}/ncbi_dataset/data/{params.assembly_id}/protein.fa
{params.dir2}
{params.species_name}/{params.species_name}_protein.faa; \
rm -r {params.dir1}{params.species_name} \
```

`Output` : "data/ncbi/Athaliana/Athaliana_genomic.gff" ; "data/ncbi/Athaliana/Athaliana_protein.faa"; "data/ncbi/Athaliana/Athaliana_cds_from_genomic.fna".

Preprocessing the gff file (default)

`Purpose` : Create a mockgff from the gff3 which can be recognize by McscanX

> ⓘ Note
>
> Since the required input .gff file for mcscanx is nether gff3 nor bed file format, for simplity, call it mockgff file

> 💡 Tip
>
> If the mkGFF3.pl does not work on your gff3 file due to the format of naming, there are other ways/options to generate the mockgff, check the next rules and substitue the mockgff with the one works.

> ⚠ Warning

> The mkGFF3.pl was adopted from MCScanX_protocol which is not exactly same (Wang, Yupeng, et al. Nature Protocols 19.7 (2024): 2206-2229.)

`scripts` :

```
mkdir -p {params.dir};\
curl -OJX \
GET "{params.link}"; \
mv {params.file} {params.dir} \
```

`Input` : data/ncbi/Athaliana/Athaliana_genomic.gff

```
##gff-version 3
#!gff-spec-version 1.21
#!processor NCBI annotwriter
#!genome-build TAIR10.1
#!genome-build-accession NCBI_Assembly:GCF_000001735.4
#!annotation-source TAIR and Araport
##sequence-region NC_003070.9 1 30427671
##species https://www.ncbi.nlm.nih.gov/Taxonomy/Browser/wwwtax.cgi?
id=3702
NC_003070.9     RefSeq  region  1       30427671        .       +
.
ID=NC_003070.9:1..30427671;Dbxref=taxon:3702;Name=1;chromosome=1;ecotyp
DNA
```

`Output` : data/intermediateData/Athaliana/Athaliana.gff

```
#mockgff
Athaliana1      NP_171609.1     3760    5630
Athaliana1      NP_001318899.1  6915    8666
Athaliana1      NP_001321777.1  6915    8442
Athaliana1      NP_001321775.1  6915    8442
Athaliana1      NP_001321776.1  6915    8419
```

Preprocessing the gff (option one)

`Purpose` : This rule uses the gff2bed tool to convert gff to bed for easier parsing (the mockgff file)

> ⚠ Warning

> To run this rule, users will need uncomment the lines in the workflow/snakefile_part1 file

```
        #       expand("data/intermediateData/{name}/{name}.gff-option_one",
        #               name = config['names']),
```

`scripts`:

```
cat {input.gff} \
| grep -v '^#' \
| awk '$3 == "gene"' \
| gff2bed \
| awk 'BEGIN {{OFS="\t"}} {{print $1,$4,$2,$3}}' \
> {output.mockgff} \
```

`Output`: data/intermediateData/Athaliana/Athaliana.gff-option_one

Preprocessing the gff (option Two)

`Purpose`: This rule can make use of the XX.feature_table.txt from NCBI to generate the mockgff for McScanX as input file.

> **Note**
>
> user will need to put the "XX.feature_table.txt" in the config.yaml file.

```
# Example of the XX.feature_table.txt:
link:
https://ftp.ncbi.nlm.nih.gov/genomes/all/GCF/000/002/595/GCF_000002595
```

`scripts`:

```
sed 1d {input.feature_table} \
|grep 'mRNA' \
|awk -F'\t' '$13!=""{{print $7"\t"$13"\t"$8"\t"$9}}' \
> {output.mockgff} \
```

`Output`: data/intermediateData/Athaliana/Athaliana.gff-option_two

> **Warning**

> To run this rule, user will need uncommenting the lines in the snakefile_part1

```
        # Other ways to yield the mockgff: featuretable_to_mockgff
        #       expand("data/intermediateData/{name}/{name}.gff-option_two",
        #               name = config['names']),
```

Prepare the primary protein for the input file

`Purpose`: This rule is the preprocessing step for extracting the longest transcript encoding for each gene, and use the primary protein for the rest of analysis.

> Note
>
> Due to alternative splicing, the mRNA isoform/transcript can have different lengths, which encoding the protein product with different ID but from same gene. This step is to minimize the misprediction of gene duplicates for those proteins encoded by alternative splicing transcitps having similiar fucntional domains.

`scripts`:

```
        python3 {params.dir1}/isoform2one.py {input.feature_table} {output}; \
        awk '{{print $1}}' {input.protein} \
```

`Output`: "data/ncbi/{name}_primary/{name}_protein.list", "data/ncbi/{name}_primary/{name}_protein.faa",

```
# Athaliana_protein.list
NP_171609.1
NP_001321775.1
NP_171611.1
NP_171612.1
NP_171613.1
NP_001320628.
```

> Note

> There are rare cases for NCBI without feature table to download (e.g., https://ftp.ncbi.nlm.nih.gov/genomes/all/GCF/000/001/735/GCF_000001735.4_TAIR10.1/GCF_000001735.4_TAIR10.1_feature_table.txt.gz). Users can prepare primary protein gene list - "XX_protein.list" from NCBI website manually, For example, the proteins column for Chlamydomonas reinhardtii: https://www.ncbi.nlm.nih.gov/datasets/gene/GCF_000002595.2/?gene_type=protein-coding

Prepare the cds file for calculating the Ka/ks ratio

`Purpose` : This rule is preprocessing step for running the McScanX with input data from genomic cds

> ⚠ Warning
>
> The mkCD.pl was adopted from MCScanX_protocol which is not exactly the same (Wang, Yupeng, et al. Nature Protocols 19.7 (2024): 2206-2229.)

`scripts` :

```
perl {params.dir2}/mkCD.pl {params.dir3} {params.species_name} \
```

`Output` : data/intermediateData/Athaliana/Athaliana.cds

```
>NP_171609.1
ATGGAGGATCAAGTTGGGTTTGGGTTCCGTCCGAACGACGAGGAGCTCGTTGGTCACTATCTCCGTAACA
AAACACTAGCCGCGACGTTGAAGTAGCCATCAGCGAGGTCAACATCTGTAGCTACGATCCTTGGAACTTG
```

Diamond_db_mcscanx

`Purpose` : This rule builds diamond database for blasting the protein sequence

`scripts` :

```
        mkdir -p {params.dir1}; \
        diamond makedb \
                --in {params.protein} \
                -d {params.db_name_dir} \
```

`Output` : data/ncbiDB/Athaliana.dmnd

Diamond_blast_mcscanx

`Purpose`: This rule runs diamond blastp for the protein sequences against themselves (blastp all vs all)

> ⓘ Note
>
> --max-target-seqs parameter will impact how many candidate duplicates will be detected `scripts`:

```
                  diamond blastp \
                  -d {params.db_name_dir} \
                  -q {params.protein} \
                  -o {output} \
                  -e 1e-10 \
                  -f 6 \
                  -p {threads} \
                  --sensitive \
                  --max-target-seqs 5 \
```

`Output`: data/intermediateData/Athaliana/Athaliana.blast

```
NP_001030613.1  NP_001030613.1  100     596     0       0       1
596     1       596     0.0     1155
NP_001030613.1  NP_001327195.1  100     583     0       0       1
583     47      629     0.0     1132
NP_001030613.1  NP_186759.2     100     583     0       0       1
583     1       583     0.0     1132
```

DupGen_finder_diamond_outgroup

`Purpose`: This rule runs diamond blastp for the species against the outgroup species

> ⓘ Note
>
> The outgroup species in the config.yaml file is used for cross-genome comparison, which is useful for suggesting other types of duplicates. `scripts`:

```
                mkdir -p {params.dir}; \
                diamond blastp \
                -d {params.db_name_dir} \
                -q {params.protein} \
                -o {params.out_name} \
                -e 1e-10 \
                -f 6 \
                -p {threads} \
                --sensitive \
                --max-target-seqs 5 \
```

`Output` : directory("data/intermediateData/Athaliana_DupGen_finder/")

```
#Athaliana_Creinhardtii.blast
NP_001030613.1	XP_042921808.1	25.3	400	274	7	198
573	492	890	1.12e-28	122
NP_001030613.1	XP_042921809.1	25.6	312	224	4	272
576	866	1176	3.84e-28	120
NP_001030613.1	XP_001702326.1	25.4	307	222	5	272
573	775	1079	1.12e-26	115
NP_001030613.1	XP_042920827.1	25.2	306	224	3	272
573	938	1242	6.41e-26	113
```

Text S3. Snakefile_part2

Prepare the gff for DupGen_finder

`Purpose` : This rule merges the gff files from species and the outgroup species into one (e.g., Athaliana_Creinhardtii.gff)

`scripts` :

```
        cp {input.gff} {params.dir2}/{params.species_name}.gff; \
        cp {params.dir1}/{params.species_name}.blast
{params.dir2}/{params.species_name}.blast; \
        cp {params.dir3}/{params.outgroup_name}.gff
{params.dir2}/{params.outgroup_name}.gff; \
        cp {params.dir3}/{params.outgroup_name}.blast
{params.dir2}/{params.outgroup_name}.blast; \
        cp {params.dir5}/{params.out_name}.blast
{params.dir2}/{params.out_name}.blast; \
        cat {params.dir1}/{params.species_name}.gff
{params.dir2}/{params.outgroup_name}.gff \
> {params.dir2}/{params.out_name}.gff \
```

`Output` : data/DupGen_finder/Athaliana_data/Athaliana_Creinhardtii.gff

```
Athaliana5      NP_201563.1     26964891        26965720
Athaliana5      NP_201564.1     26967535        26969306
Athaliana5      NP_201565.1     26969546        26970548
Creinhardtii1   XP_042927982.1  19163   19948
Creinhardtii1   XP_042927983.1  20398   22476
Creinhardtii1   XP_042927984.1  24126   29577
```

DupGen_finder

`Purpose` : This rule can identify different modes of duplicated gene pairs (detecting WGD > tandem > proximal > transposed > dispersed duplicates)

> ⚠ Warning
>
> The DupGen_finder.pl was adopted from DupGen_finder which is not exactly same (Qiao, Xin, et al. Genome biology 20 (2019): 1-23; Wang, Yupeng, et al. Nucleic acids research 40.7 (2012): e49-e49). https://github.com/qiao-xin/DupGen_finder/blob/master/DupGen_finder.pl

`scripts` :

```
        mkdir -p {params.dir5}; \
        sleep 30s; \
        perl {params.dir4}/DupGen_finder.pl \
        -i {params.dir2} \
        -t {params.species_name} \
        -c {params.outgroup_name} \
        -o {params.dir5} \
```

`Output`:

```
                dispersed =
"data/DupGen_finder/{name}_result/Athaliana.dispersed.pairs",
                proximal =
"data/DupGen_finder/{name}_result/Athaliana.proximal.pairs",
                transposed =
"data/DupGen_finder/{name}_result/Athaliana.transposed.pairs",
                WGD =
"data/DupGen_finder/Athaliana_result/Athaliana.wgd.pairs",
                collinearity =
"data/DupGen_finder/Athaliana_result/Athaliana.collinearity"
```

```
#tandem =
"data/DupGen_finder/Athaliana_result/Athaliana.tandem.pairs",
Duplicate 1     Location        Duplicate 2     Location        E-
value
NP_001030614.1  Athaliana3:94343        NP_001327831.1
Athaliana3:94343        0.0
NP_001030615.2  Athaliana3:121120       NP_001078086.1
Athaliana3:122140       5.38e-29
NP_001030616.1  Athaliana3:127557       NP_186783.1
Athaliana3:127557       1.06e-175
NP_001030617.1  Athaliana3:137772       NP_186785.1
Athaliana3:137772       1.61e-222
...
# WGD > tandem (TD) > proximal(PD) > transposed(TRD) > dispersed
duplicates(DD)
Types    NO. of gene pairs
WGD-pairs    4254
TD-pairs    2063
PD-pairs    870
TRD-pairs    4861
DD-pairs    16208
```

DupGen_finder_unique

`Purpose` : This rule can remove redundant duplicate genes for different modes of duplicated gene pairs (WGD > tandem > proximal > transposed > dispersed duplicates).

> **Note**
>
> To eliminate redundant duplicate genes among different modes,DupGen_finder-unique.pl is a stricter version of DupGen_finder, which can assign each duplicate gene to an unique mode after all of the duplicated gene pairs were classified into different gene duplication types.

> **Warning**
>
> The DupGen_finder-unique.pl was adopted from DupGen_finder which is not exactly same (Qiao, Xin, et al. Genome biology 20 (2019): 1-23; Wang, Yupeng, et al. Nucleic acids research 40.7 (2012): e49-e49). https://github.com/qiao-xin/DupGen_finder/blob/master/DupGen_finder-unique.pl

`scripts` :

```
        perl {params.dir4}/DupGen_finder-unique.pl \
        -i {params.dir2} \
        -t {params.species_name} \
        -c {params.outgroup_name} \
        -o {params.dir5} \
```

`Output` :

```
                tandem =
"data/DupGen_finder/Athaliana_result_uniq/Athaliana.tandem.genes-
unique",
                dispersed =
"data/DupGen_finder/Athaliana_result_uniq/Athaliana.dispersed.genes-
unique",
                proximal =
"data/DupGen_finder/Athaliana_result_uniq/Athaliana.proximal.genes-
unique",
                transposed =
"data/DupGen_finder/Athaliana_result_uniq/Athalianatransposed.genes-
unique",
                WGD =
"data/DupGen_finder/Athaliana_result_uniq/Athaliana.wgd.genes-
unique",
                gff =
"data/DupGen_finder/Athaliana_result_uniq/Athaliana.gff.sorted"
```

```
#Athaliana.all.genes-unique
Duplicate       chrom   Location
NP_171609.1     Athaliana1      3760
NP_001318899.1  Athaliana1      6915
NP_001321775.1  Athaliana1      6915
NP_001321776.1  Athaliana1      6915
NP_001321777.1  Athaliana1      6915
NP_001030923.1  Athaliana1      7315
NP_001321778.1  Athaliana1      7315

# WGD > tandem (TD) > proximal(PD) > transposed(TRD) > dispersed
duplicates(DD)
Types   NO. of gene pairs
WGD-pairs    4254
TD-pairs    1563
PD-pairs    554
TRD-pairs    4861
DD-pairs    7160
```

Calculating ka_and_ks values from the duplicates pairs

`Purpose`: This rule can run the PAML package (Yang, Ziheng.Molecular biology and evolution 24.8 (2007): 1586-1591.) to calculate the kaks for the gene pairs.

> ⚠ Warning

> The add_ka_and_ks_to_collinearity_Yn00.pl was adopted from DupGen_finder which is not exactly same (Qiao, Xin, et al. Genome biology 20 (2019): 1-23; Wang, Yupeng, et al. Nucleic acids research 40.7 (2012): e49-e49). https://github.com/qiao-xin/Scripts_for_GB/tree/master/identify_Ks_peaks_by_fitting_GMM

`scripts`:

```
        cp {params.dir1}/{params.species_name}.cds
{params.dir5}/{params.species_name}.cds; \
        sleep 30s; \
        perl {params.dir4}/add_ka_and_ks_to_collinearity_Yn00.pl \
        -i {input} \
        -d {params.dir5}/{params.species_name}.cds \
        -o {output} \
```

`Output`: data/DupGen_finder/Athaliana_result/Athaliana.kaks

```
NP_001321164.1  NP_001185394.1  0.2543  0.7844  0.3242    2e-73
NP_001322884.1  NP_001320573.1  0.2128  0.9219  0.2308        0
NP_564051.1     NP_001323057.1  0.1533  0.8397  0.1826    4e-62
NP_173281.1     NP_565075.1     0.1210  0.8860  0.1365        0
NP_564052.1     NP_001322804.1  0.1003  0.5209  0.1926   2e-270
NP_173285.2     NP_001322762.1  0.0908  0.9425  0.0963   1e-281
NP_173286.2     NP_683494.2     0.2178  1.3273  0.1641   4e-131
NP_173289.1     NP_177545.1     0.0523  0.9223  0.0567   3e-147
```

> ⓘ Note
>
> Due to the perl script: add_ka_and_ks_to_collinearity_Yn00.pl, which may have some temporary files left in main dir, which can be safefly removed.

```
#       rm -p *.aln; \
#       rm -p *.cds; \
#       rm -p *.dnd; \
#       rm -p *.pro; \
```

Adding_Ka_Ks_into_collinearity

`Purpose`: This rule is to preprocess the XX.collinearity and XX.kaks file for the next step

`scripts`:

```
        mkdir -p {params.dir}; \
        cp {input.col}
{params.dir}/{params.species_name}.collinearity; \
        awk -F'\t' '{{print $2"\t"$3"\t"$5"\t"$6"\t"$7"\t"$4}}'
{input.kaks} \
|grep -v -e '^[[:space:]]*$' \
> {output[0]}
```

`Output` : data/DupGen_finder/Athaliana_result_kaks/Athaliana.collinearity

```
############### Parameters ###############
# MATCH_SCORE: 50
# MATCH_SIZE: 5
# GAP_PENALTY: -1
# OVERLAP_WINDOW: 5
# E_VALUE: 1e-05
# MAX GAPS: 25
############### Statistics ###############
# Number of collinear genes: 6451, Percentage: 13.40
# Number of all genes: 48147
##########################################
## Alignment 0: score=4086.0 e_value=0 N=91 Athaliana1&Athaliana1
plus
  0-  0:        NP_001321164.1  NP_001185394.1    2e-73
  0-  1:        NP_001322884.1  NP_001320573.1        0
  0-  2:        NP_564051.1     NP_001323057.1    4e-62
```

Adding_Ka_Ks_into_collinearity2

`Purpose` : This rule can add Ka, Ks, Ka/Ks values into Athaliana.collinearity by using Athaliana.kaks as input, and produce one output file: Athaliana.collinearity.kaks

> ⚠ Warning
>
> The add_ka_ks_to_collinearity_file.pl was adopted from DupGen_finder which is not exactly the same (Qiao, Xin, et al. Genome biology 20 (2019): 1-23; Wang, Yupeng, et al. Nucleic acids research 40.7 (2012): e49-e49). https://github.com/qiao-xin/Scripts_for_GB/tree/master/identify_Ks_peaks_by_fitting_GMM

`scripts` :

```
perl {params.dir1}/add_ka_ks_to_collinearity_file.pl
{params.dir2}/{params.species_name} \
```

`Output` : data/DupGen_finder/Athaliana_result_kaks/Athaliana.collinearity.kaks

```
############### Parameters ###############
# MATCH_SCORE: 50
# MATCH_SIZE: 5
# GAP_PENALTY: -1
# OVERLAP_WINDOW: 5
# E_VALUE: 1e-05
# MAX GAPS: 25
############### Statistics ###############
# Number of collinear genes: 6451, Percentage: 13.40
# Number of all genes: 48147
##########################################
## Alignment 0: score=4086.0 e_value=0 N=91 Athaliana1&Athaliana1
plus
  0-  0:        NP_001321164.1  NP_001185394.1    2e-73 0.2543
0.7844  0.3242
  0-  1:        NP_001322884.1  NP_001320573.1        0 0.2128
0.9219  0.2308
  0-  2:        NP_564051.1     NP_001323057.1    4e-62 0.1533
0.8397  0.1826
  0-  3:        NP_173281.1     NP_565075.1           0 0.1210
0.8860  0.1365
```

Calculating_Ks_syntenic_block

`Purpose` : This rule produces one output file: Athaliana.synteny.blocks.ks.info, which contains average Ks values for gene pairs contained in each syntenic block.

> ⚠ Warning
>
> The compute_ks_for_synteny_blocks.pl was adopted from DupGen_finder which is not exactly same (Qiao, Xin, et al. Genome biology 20 (2019): 1-23; Wang, Yupeng, et al. Nucleic acids research 40.7 (2012): e49-e49). https://github.com/qiao-xin/Scripts_for_GB/tree/master/identify_Ks_peaks_by_fitting_GMM

`scripts` :

```
        perl {params.dir1}/compute_ks_for_synteny_blocks.pl {input};
\
        cp {params.species_name}.synteny.blocks.ks.info {output}; \
        rm {params.species_name}.synteny.blocks.ks.info \
```

`Output` : data/DupGen_finder/Athaliana_result_kaks/Athaliana.synteny.blocks.ks.info

```
Blocks ID       Location        Block Size      Average Ks      e-
value   Score   Orientation
Alignment169    Athaliana5&Athaliana5   16      0.94783125
2.9e-41 719.0   plus
Alignment105    Athaliana2&Athaliana5   10      2.41932 8.7e-21
430.0   minus
Alignment159    Athaliana4&Athaliana5   6       1.43243333333333
8.6e-09 262.0   plus
Alignment9      Athaliana1&Athaliana1   14      1.66228571428571
8.2e-34 640.0   plus
Alignment148    Athaliana3&Athaliana5   6       0.922966666666667
0       264.0   minus
Alignment119    Athaliana3&Athaliana5   95      0.920378494623656
0       4241.0  plus
```

Estimating Ks peaks from Ks distribution

`Purpose` : This rule can create the Ks distribution of Ks values of syntenic blocks within the genome

> ⚠ Warning
>
> The plot_syntenic_blocks_ks_distri.py was adopted from DupGen_finder which is not exactly same (Qiao, Xin, et al. Genome biology 20 (2019): 1-23; Wang, Yupeng, et al. Nucleic acids research 40.7 (2012): e49-e49). https://github.com/qiao-xin/Scripts_for_GB/tree/master/identify_Ks_peaks_by_fitting_GMM

> ⓘ Note
>
> The parameter 'Components' can indicate the number of the mixture components, which represents the number of Ks peak.

`scripts` :

```
perl {params.dir1}/plot_syntenic_blocks_ks_distri.py {input}
{params.components} {params.dir2}/{params.species_name} \
```

`Output` : data/DupGen_finder/Athaliana_result_kaks/Athaliana.synteny.blocks.ks.distri.pdf

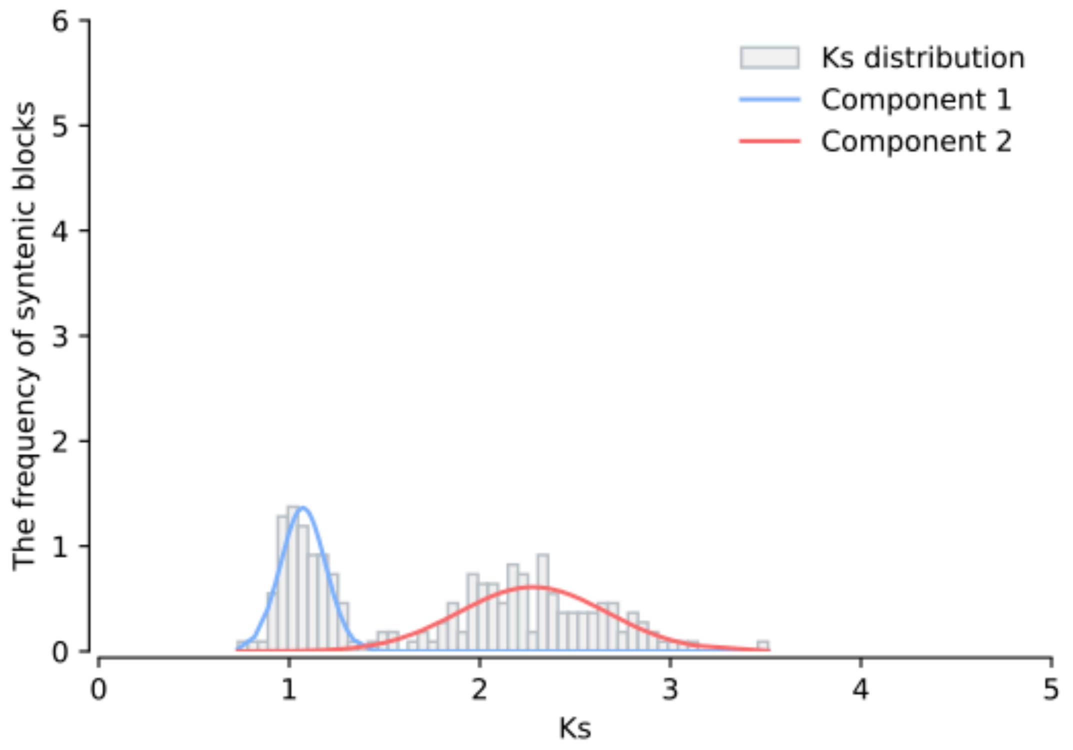

Text S4. Snakefile_part3

Prepare_hsdfinder_inputs

`Purpose` : This step is to generate a protein fasta file with short header line.

`scripts` :

```
        mkdir -p {params.dir1}; \
        awk '{{print $1}}' {input.protein} \
```

`Output` : data/hsdfinder/Athaliana.fa

```
>NP_001030613.1
MLLSALLTSVGINLGLCFLFFTLYSILRKQPSNVTVYGPRLVKKDGKSQQSNEFNLERLLPTAGWVKRAL[illegible]
LGLDALVFIRVFVFSIRVFSFASVVGIFILLPVNYMGTEFEEFFDLPKKSMDNFSISNVNDGSNKLWIHF[illegible]
```

Prepare_duplication_all_list

`Purpose`: acquire a list of unique gene duplicates for all duplication modes, and then furtherly refined by hsdfinder to acquire higly similiar duplicates (HSDs)

`scripts`:

```
        mkdir -p {params.dir1}; \
        cp {input.protein} {params.all}; \
        sed -e '1i\Duplicate\tchrom\tLocation' {input.gff_sorted} \
> {output.dup_mock_genes_unique} \
```

`Output`: "data/DupGen_finder/Athaliana_result_uniq/Athaliana.all.genes-unique"

```
Duplicate       chrom   Location
NP_171609.1     Athaliana1      3760
NP_001318899.1  Athaliana1      6915
NP_001321775.1  Athaliana1      6915
NP_001321776.1  Athaliana1      6915
NP_001321777.1  Athaliana1      6915
NP_001030923.1  Athaliana1      7315
NP_001321778.1  Athaliana1      7315
NP_001322175.1  Athaliana1      11864
```

Prepare hsdfinder diamond database

`Purpose`: This rule creates diamond database for the next step of all vs all blast.

`scripts`:

```
        diamond makedb \
                --in {input.protein} \
                -d {params.db_name_dir} \
```

`Output`: results/Athaliana_tandem/Athaliana_tandem.dmnd

Prepare the protein for hsdfinder

`Purpose`: The rule runs the diamond blastp all vs all for each type of gene duplicates (output a tabular format blastp result)

> ⓘ Note

> Since amino acid substitutions occur less frequently than nucleotide substitutions, the sequence alignments are thereby generally evaluated by amino acid sequences instead of nucleotides, which allows a greater sensitivity (Koonin and Galperin, 2002).

`scripts` :

```
                diamond blastp \
                -d {params.db_name_dir} \
                -q {input.protein} \
                -o {output} \
                -e 1e-10 \
                -f 6 \
                -p {threads} \
                --sensitive \
```

`Output` : results/Athaliana_tandem/diamond/Athaliana_tandem.txt

```
NP_001030613.1  NP_001030613.1  100     596     0       0       1
596     1       596     0.0     1155
NP_001030613.1  NP_001327195.1  100     583     0       0       1
583     47      629     0.0     1132
NP_001030613.1  NP_186759.2     100     583     0       0       1
583     1       583     0.0     1132
NP_001030613.1  NP_001327194.1  100     468     0       0       116
583     1       468     0.0     917
```

> ⓘ Note
>
> So we chose the protein sequence to do the Diamond blastp all-against-all search (Buchfink et al., 2015) (defaulted parameters: E-value cut-off ≤ 1e-10, blastp -outfmt 6 etc.).

interproscan (dependencies)

`Purpose` : This step is to generate the protein annotaion file by running interproscan search.

> ⚠ Warning

> The InterProScan (Quevillon et al., 2005, Mitchell et al., 2019) and KEGG (Kanehisa and Goto, 2000b) are the only two dependencies without integrating into the HSDSnake pipeline due to the lack of Conda environment (the latest InterProScan Conda package of 5.59 fails in SnakeMake) and the limitation to web-only access in KEGG, such as BlastKOALA (Kanehisa et al., 2016)).

> **Tip**
>
> It is straightforward to generate the InterProScan output by either checking the respective ReadMe file or following the protocol at Step 6-9.

`scripts` : shell script

```
/interproscan.sh -i proteins_of_your_genome.fasta -f tsv -dp
```

`Output` : data/Athaliana.interproscan.tsv

> **Warning**
>
> To make sure the protein sequecne is consistent under different analysis, please use the preprocessed fasta to submit for the InterProScan search, i.e., the file under the directory: data/hsdfinder/Athaliana_all/Athaliana.all.fa

kegg blastkoala (dependencies)

`Purpose` : This step is to generate a KEGG functional category file.

`scripts` : local submission

> **Warning**
>
> To make sure the protein sequecne is consistent under different analysis, please use the preprocessed fasta to submit for the KEGG Blastkoala search, i.e., the file under the directory: data/hsdfinder/Athaliana_all/Athaliana.all.fa

`Output` :data/Athaliana.interproscan.ko.txt

> **Tip**

> It is straightforward to generate the kegg blastkoala output by either checking the respective website or following the protocol at Step 17-20.

HSDfinder preprocess

`Purpose` : This step is to debug the previous raised potential issue with using HSDFinder

`scripts` : shell script

```
        (awk '/^>/{{if (l!="") print l; print; l=0; next}}
{{l+=length($0)}}END{{print l}}' \
                {input.protein} \
                |paste - - \
                |sed 's/>//g' \
                |awk -F'\t' '{{print $1"\t"$1"\t"100"\t"$2}}' \
                |cat ;\
                cat {input.tabular}) \
                |cat \
```

`Output` : results/Athaliana_tandem/diamond/Athaliana_tandem.preprocess.txt

hsdfinder

`Purpose` : This step is the main script to generate HSDs with different thresholds.

`scripts` : shell script

```
        hsdfinder \
                -i {input.tabular} \
                -p {params.identity} \
                -l {params.variance} \
                -f {input.Interproscan} \
                -t Pfam \
                -o {output} \
```

`Output` : results/Athaliana_tandem/hsdfinder/Athaliana_tandem.90_10.txt

```
NP_171636.1     NP_171636.1; NP_001154299.1     270; 270        Pfam
PF01967;        MoaC family;    2.9E-53;        IPR002820;
Molybdopterin cofactor biosynthesis C (MoaC) domain;
NP_171639.3     NP_171639.3; NP_001321495.1; NP_001184884.1
1797; 1789; 1787        Pfam    PF12807, PF15044, PF13424; ;
Translation initiation factor eIF3 subunit 135, Mitochondrial
function, CLU-N-term, Tetratricopeptide repeat; ;       1.9E-22,
1.5E-8, 2.8E-13; ;      IPR033646, IPR028275, -; ;      CLU central
domain, Clustered mitochondria protein, N-terminal, -; ;
```

```
Column explanation:
1. Highly Similar Duplicates (HSDs) identifiers: The first gene
model of the duplicate gene copies is used as the HSD identifers in
default. (e.g. g735.t1)
2. Duplicate gene copies (within 10 amino acids, ≥90% pairwise
identities)(e.g. g735.t1; g741.t1; g8053.t1)
3. Amino acid length of duplicate gene copies (aa)(e.g. 744; 744;
747)
4. Pfam identifier (e.g. PF11999; PF11999; PF11999)
5. Analysis (e.g. Pfam / PRINTS / Gene3D)
6. Pfam Description (e.g. Protein of unknown function (DUF3494);
Protein of unknown function (DUF3494); Protein of unknown function
(DUF3494))
7. InterPro Entry Identifier (e.g. IPR021884; IPR021884; IPR021884)
8. InterPro Entry Description (e.g. Ice-binding protein-like ; Ice-
binding protein-like ; Ice-binding protein-like)
```

> **Tip**
>
> For the specific usage of HSDFinder tool, please find here

kegg category

`Purpose` : This step is to apply KEGG functional category on the detected HSDs.

`scripts` : shell script

```
        python3 $PWD{params.dir}HSD_to_KEGG.py \
                -i {input.HSD_result} \
                -k {input.KEGG} \
                -n {params.species_name}\
                -o {output} \
```

`Output` : results/Athaliana_tandem/kegg/Athaliana_tandem.90_10.kegg.txt

```
09101 Carbohydrate metabolism   00010 Glycolysis / Gluconeogenesis
[PATH:ko00010]  K01810  GPI, pgi; glucose-6-phosphate isomerase
[EC:5.3.1.9]    NP_001332180.1  Athaliana_tandem        NP_199088.1
1
09101 Carbohydrate metabolism   00010 Glycolysis / Gluconeogenesis
[PATH:ko00010]  K00850  pfkA, PFK; 6-phosphofructokinase 1
[EC:2.7.1.11]   NP_200966.2     Athaliana_tandem        NP_200966.2
1
09101 Carbohydrate metabolism   00010 Glycolysis / Gluconeogenesis
[PATH:ko00010]  K03841  FBP, fbp; fructose-1,6-bisphosphatase I
[EC:3.1.3.11]   NP_190973.1     Athaliana_tandem        NP_190973.1
1
```

```
Column explanation:

   1. The identifier (e.g. 0)
   2. Pathway category1 (e.g. 09101 Carbohydrate metabolism)
   3. Pathway category2 (e.g. 00010 Glycolysis / Gluconeogenesis
[PATH:ko00010])
   4. KEGG ko_id (e.g. K13979)
   5. function (e.g. yahK; alcohol dehydrogenase (NAP+))
   6. species_name (e.g. UWO241) Chlamydomonas sp. UWO241
   7. hsds_id (e.g. g1713.t1)
   8. hsds_num (e.g. 1)
```

> **Tip**
>
> For the specific usage of HSD_to_KEGG.py, please read here

hsdecipher statistcs

`Purpose` : This step is to calculate the statistics of HSDs via using a variety of HSDFinder thresholds. https://github.com/zx0223winner/HSDecipher#2-whats-hsdecipher

`scripts` : shell script

```
        python3 $PWD{params.dir}HSD_statistics.py \
                {params.HSD_dir} \
                {params.HSD_file_format} \
                {output} \
```

`Output` : results/Athaliana_tandem/hsdecipher/stats/Athaliana_tandem.stat.txt

```
File_name       Candidate_HSDs# Non-redundant_gene_copies#
Gene_copies#    True_HSDs#      Space#  Incomplete_HSDs#
Capturing_value Performance_score
Athaliana_tandem.50_10  6441    18955   19115   5338    6033    1103
82.88   5.2
Athaliana_tandem.50_100 6904    27790   28570   5341    6323    1563
77.36   3.79
Athaliana_tandem.50_30  6926    23587   24220   5571    6451    1355
80.44   4.46
Athaliana_tandem.50_50  6976    25642   26698   5514    6466    1462
79.04   4.12
```

> **Tip**
>
> For the specific usage of HSD_statistics.py, please read here

hsdecipher category

`Purpose` : This step is to count the number of HSD with two, three, and more than four categories, which is helpful to evaluate the distribution of groups in HSDs.

`scripts` : shell script

```
        python3 $PWD{params.dir}HSD_categories.py \
                {params.HSD_dir} \
                {params.HSD_file_format} \
                {output} \
```

`Output` : results/Athaliana_tandem/hsdecipher/stats/Athaliana_tandem.category.txt

```
File_name	2-group_HSDs#	3-group_HSDs#	>=4-group_HSDs#
Athaliana_tandem.50_10	3846	1302	1293
Athaliana_tandem.50_100	3204	1324	2376
Athaliana_tandem.50_30	3639	1388	1899
Athaliana_tandem.50_50	3480	1368	2128
Athaliana_tandem.50_70	3344	1329	2263
Athaliana_tandem.60_10	4003	1288	1144
```

> **Note**
>
> The HSD_categories.py can be run individually for the folder results/heatmap_inter/HSD/ to acquire the overall comparison of HSDs categories among different gene duplicate modes (e.g.,Athaliana_tandem.batch_run.txt).

> **Tip**
>
> For the specific usage of HSD_categories.py, please read here

hsdecipher merge statistics

`Purpose` : This step is to merge the above analysis.

`scripts` : shell script

```
        paste -d"\t" \
        {input.stat} \
        {input.category} \
        > {output} \
```

`Output` : results/Athaliana_tandem/hsdecipher/stats/Athaliana_tandem.complete.stats.txt

```
File_name	Candidate_HSDs#	Non-redundant_gene_copies#	Gene_copies#	True_HSDs#	Space#	Incomplete_HSDs#	Capturing_value	Performance_score	File_name	2-group_HSDs#	3-group_HSDs#	>=4-group_HSDs#
Athaliana_tandem.50_10	6441	18955	19115	5338	6033	1103	82.88	5.2	Athaliana_tandem.50_10	3846	1302	1293
Athaliana_tandem.50_100	6904	27790	28570	5341	6323	1563	77.36	3.79	Athaliana_tandem.50_100	3204	1324	2376
Athaliana_tandem.50_30	6926	23587	24220	5571	6451	1355	80.44	4.46	Athaliana_tandem.50_30	3639	1388	1899
```

hsdecipher batch run

`Purpose`: This step can do a series of combination thresholds at once. To minimize the redundancy and to acquire a larger dataset of HSD candidates

`scripts`: shell script

```
        mkdir -p {params.HSD_batch_run_dir}/{params.species_name}; \
        cp {params.HSD_dir}/*
{params.HSD_batch_run_dir}/{params.species_name}/; \
        python3 $PWD{params.dir}HSD_batch_run.py \
                -i {params.HSD_batch_run_dir}; \
        rm -r {params.HSD_batch_run_dir}/{params.species_name} \
```

`Output`:results/Athaliana_tandem/hsdecipher/batch_run/Athaliana_tandem.batch_run.txt

```
NP_027726.1     NP_027726.1; NP_001077883.1; NP_001031339.1;
NP_973428.1     374; 328; 326; 326      Pfam    ; ; ;   ; ; ;   ; ;
;       ; ; ;   ; ; ;
NP_028242.1     NP_028242.1; NP_001324370.1; NP_849959.1        307;
224; 224        Pfam    ; ;     ; ;     ; ;     ; ;     ; ;
NP_029567.1     NP_029567.1; NP_001324173.1; NP_001031444.2;
NP_001189635.1; NP_001324174.1  909; 909; 911; 933; 892 Pfam    ; ;
; ;     ; ; ; ;         ; ; ; ;         ; ; ; ;         ; ; ; ;
NP_029729.1     NP_029729.1; NP_001325198.1; NP_180032.2;
NP_180712.1     219; 316; 363; 360      Pfam    ; ; ;   ; ; ;   ; ;
;       ; ; ;   ; ; ;
```

```
Column explanation:
1. Highly Similar Duplicates (HSDs) identifiers: The first gene
model of the duplicate gene copies is used as the HSD identifers in
default. (e.g. g735.t1)
2. Duplicate gene copies (within 10 amino acids, ≥90% pairwise
identities)(e.g. g735.t1; g741.t1; g8053.t1)
3. Amino acid length of duplicate gene copies (aa)(e.g. 744; 744;
747)
4. Pfam identifier (e.g. PF11999; PF11999; PF11999)
5. Analysis (e.g. Pfam / PRINTS / Gene3D)
6. Pfam Description (e.g. Protein of unknown function (DUF3494);
Protein of unknown function (DUF3494); Protein of unknown function
(DUF3494))
7. InterPro Entry Identifier (e.g. IPR021884; IPR021884; IPR021884)
8. InterPro Entry Description (e.g. Ice-binding protein-like ; Ice-
binding protein-like ; Ice-binding protein-like)
```

> Tip
>
> For the specific usage of HSD_batch_run, please read here

hsdecipher heatmap intra species

`Purpose` : This step is able to visualize the collected HSDs in a heatmap and compare the HSDs sharing the same pathway function. This can be done inta-specise and inter-speies heatmaps.

`scripts` : shell script

```
        mkdir -p {params.HSD_heatmap_dir}/{params.species_name}; \
        mkdir -p {params.ko_dir}; \
        sleep 60s; \
        cp {input.KEGG} {params.ko_dir}/; \
        cp {params.HSD_dir}/*
{params.HSD_heatmap_dir}/{params.species_name}/; \
        cp {params.batch_run}
{params.HSD_heatmap_dir}/{params.species_name}/; \
        hsdecipher \
        -f {params.HSD_heatmap_dir}/{params.species_name} \
        -k {params.ko_dir} \
        -r {params.r} \
        -c {params.c}; \
        rm -r {params.ko_dir}; \
        rm -r {params.HSD_heatmap_dir}/{params.species_name}; \
        mv {params.heatmap} {params.HSD_heatmap_dir}; \
        mv {params.tabular} {params.HSD_heatmap_dir}; \
```

`Output`: results/Athaliana_tandem/hsdecipher/heatmap/Athaliana_tandem.output_heatmap.tsv

`Output`: results/Athaliana_tandem/hsdecipher/heatmap/Athaliana_tandem.output_heatmap.eps

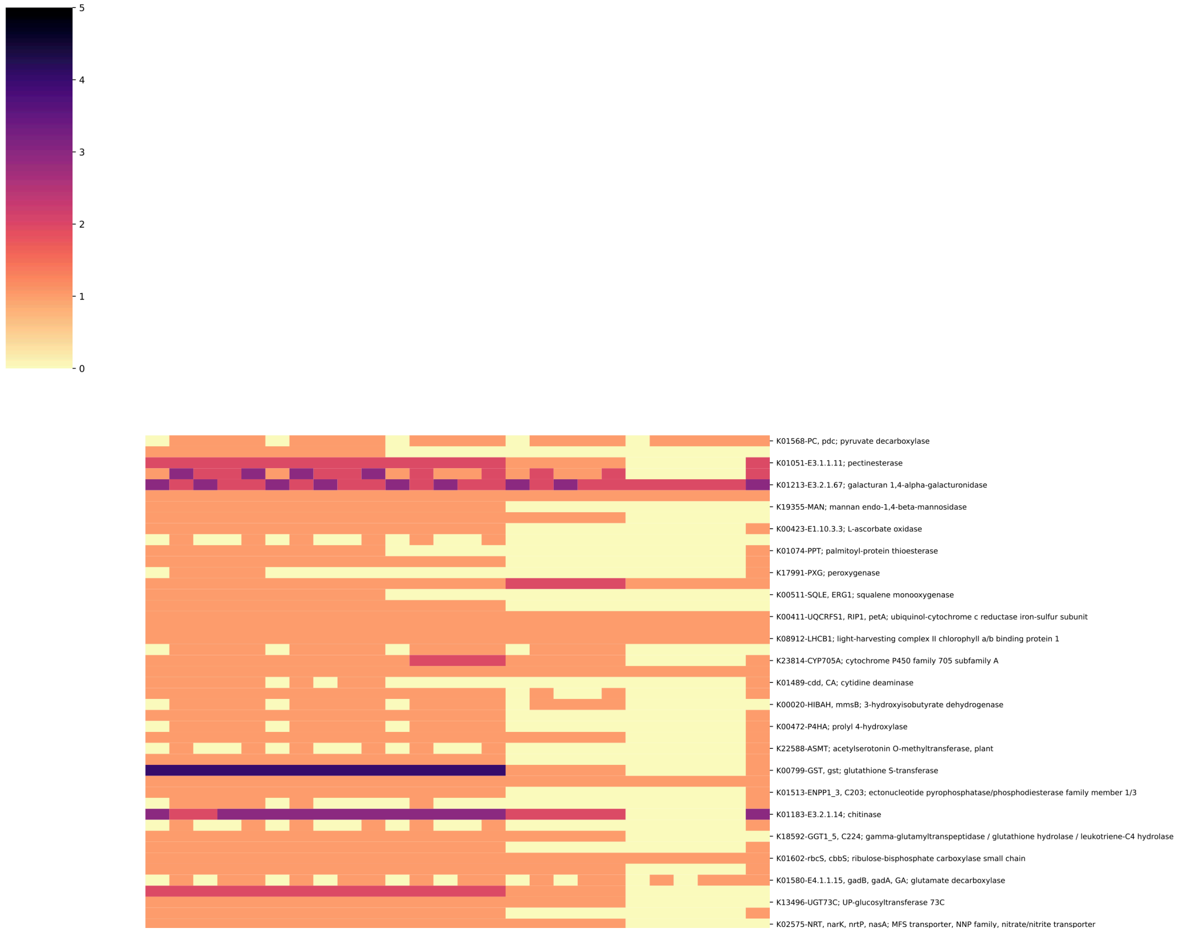

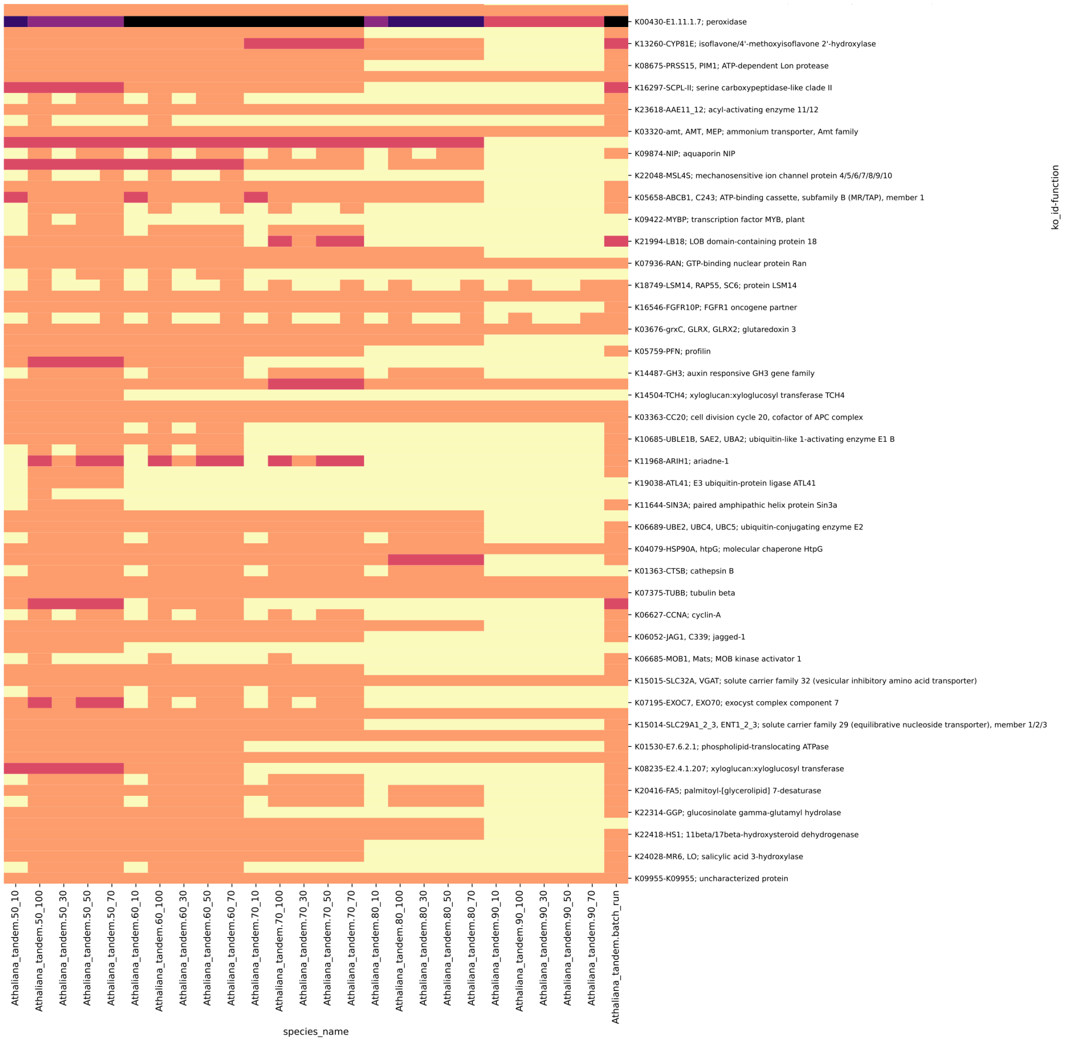

Note

The color matrix in the heatmap represent the number of HSDs being grouped together under the kegg framework.For example, the darker the color means more HSDs who shares the same KEGG ko number with each other.

Warning

To best compare the HSDs in heatmap across intra- or inter- species/genomes, the heatmap represents those KEGG ko fucntion shared by at least two comparing targets. In another words, the unique or genome-specific KEGG ko numbers will not show up in the heatmap. For more deatils, user can check the relevant output heatmap tabular file (results/heatmap_inter/HSD.output_heatmap.tsv)

Tip

[For the specific usage of HSD_heatmap.py (i.e., hsdecipher), please read here]

hsdecipher heatmap inter species

`Purpose` : This step is able to visualize the collected HSDs in a heatmap and compare the HSDs sharing the same pathway function. This can be done inta-specise and inter-speies heatmaps.

`scripts` : shell script

```
mkdir -p {params.HSD_heatmap_dir}; \
mkdir -p {params.ko_heatmap_dir}; \
sleep 30s; \
cp {params.batch_run} {params.HSD_heatmap_dir}; \
cp {input.KEGG} {params.ko_heatmap_dir}; \
hsdecipher \
-f {params.HSD_heatmap_dir}\
-k {params.ko_heatmap_dir} \
-r {params.r} \
-c {params.c}; \
mv {params.heatmap} {params.HSD_heatmap}||true; \
mv {params.tabular} {params.HSD_heatmap}||true; \
```

`Output` : results/heatmap_inter/HSD.output_heatmap.tsv `Output` : results/heatmap_inter/HSD.output_heatmap.eps

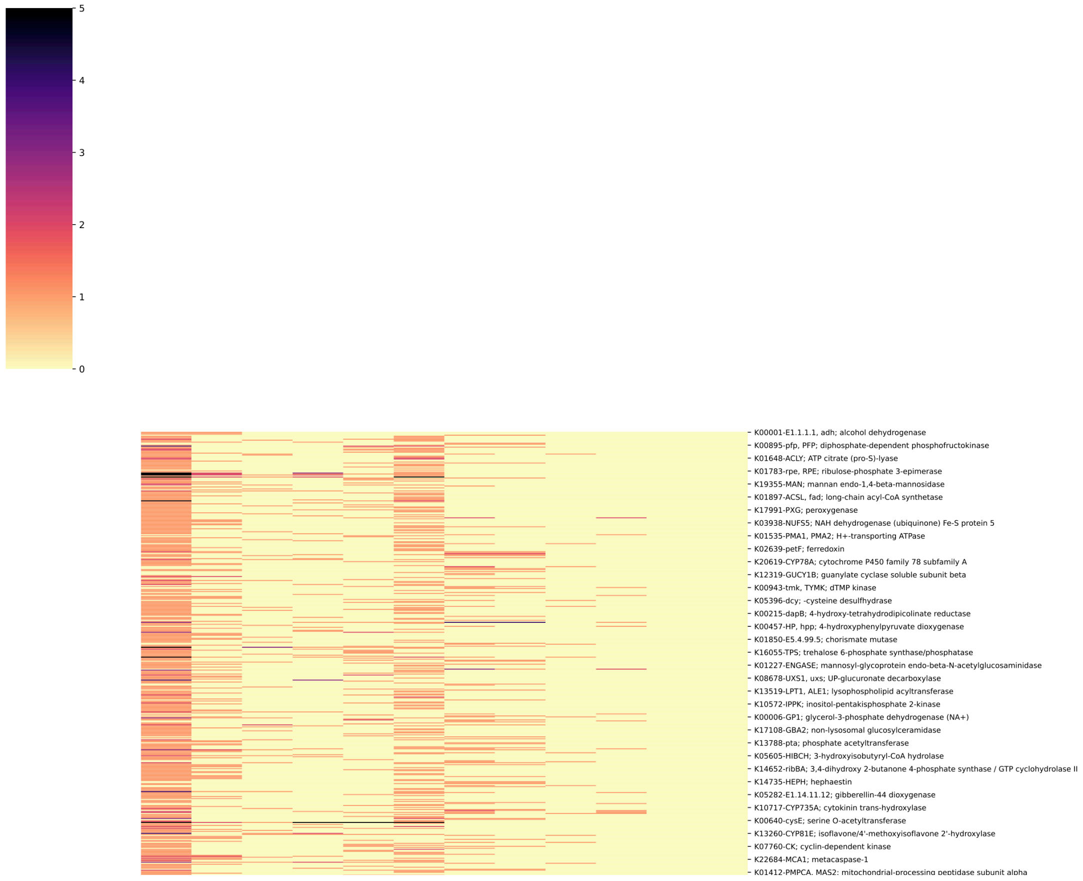

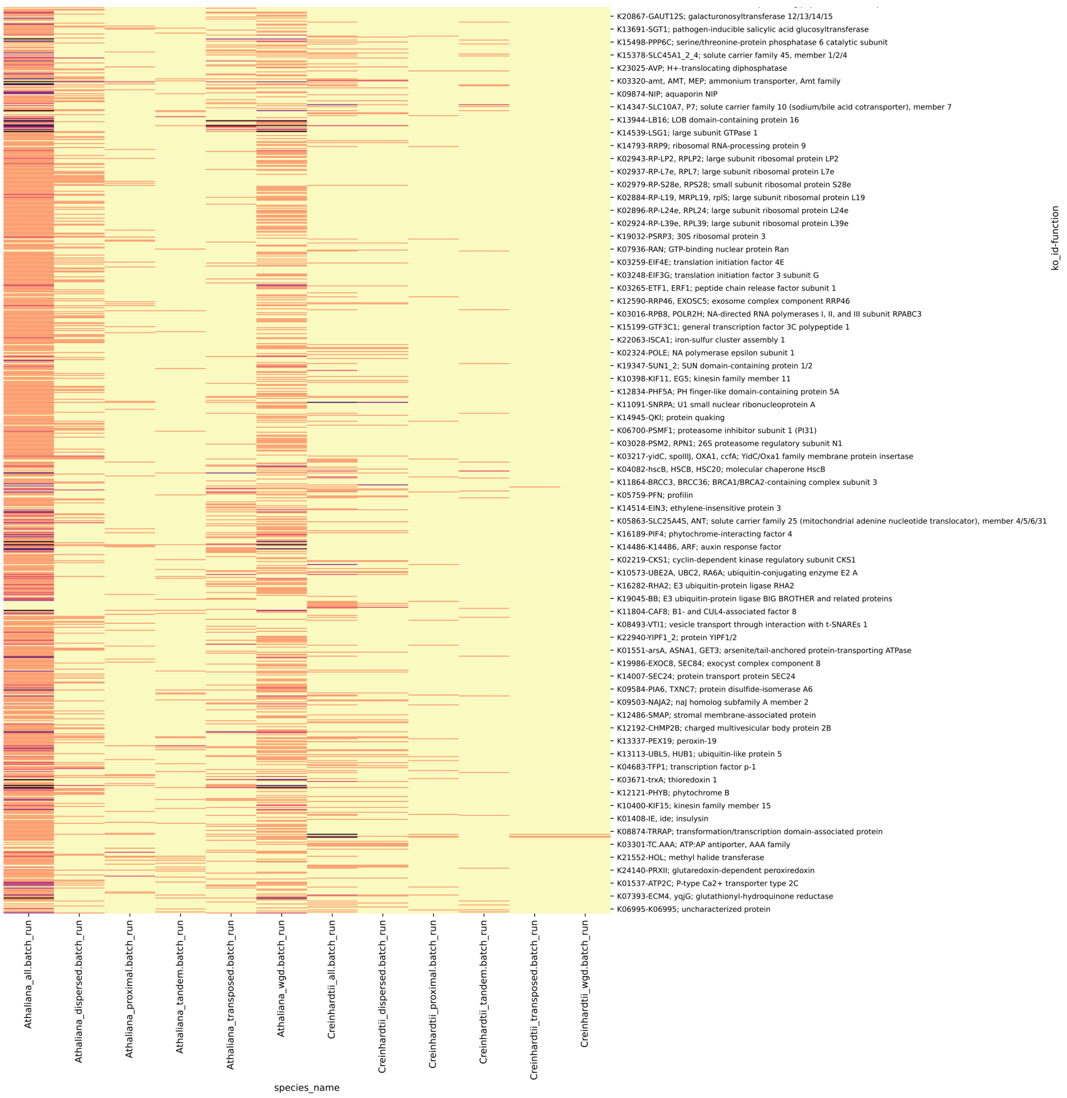

ⓘ Note

The color matrix in the heatmap represent the number of HSDs being grouped together under the kegg framework.For example, the darker the color means more HSDs who shares the same KEGG ko number with each other.

⚠ Warning

To best compare the HSDs in heatmap across intra- or inter- species/genomes, the heatmap represents those KEGG ko fucntion shared by at least two comparing targets. In another words, the unique or genome-specific KEGG ko numbers will not show up in the heatmap. For more deatils, user can check the relevant output heatmap tabular file (results/heatmap_inter/HSD.output_heatmap.tsv)

Tip

[For the specific usage of HSD_heatmap.py (i.e., hsdecipher), please read here]()

References:

McScanX_protocol: Wang, Yupeng, et al. "Detection of colinear blocks and synteny and evolutionary analyses based on utilization of MCScanX." Nature Protocols 19.7 (2024): 2206-2229.

McScanX: Wang, Yupeng, et al. "MCScanX: a toolkit for detection and evolutionary analysis of gene synteny and collinearity." Nucleic acids research 40.7 (2012): e49-e49.

DupGen_finder: Qiao, Xin, et al. "Gene duplication and evolution in recurring polyploidization–diploidization cycles in plants." Genome biology 20 (2019): 1-23.

KEGG_BlastKOALA: Kanehisa, Minoru, Yoko Sato, and Kanae Morishima. "BlastKOALA and GhostKOALA: KEGG tools for functional characterization of genome and metagenome sequences." Journal of molecular biology 428.4 (2016): 726-731.

InterProScan: Jones, Philip, et al. "InterProScan 5: genome-scale protein function classification." Bioinformatics 30.9 (2014): 1236-1240.

PAML: Yang, Ziheng. "PAML 4: phylogenetic analysis by maximum likelihood." Molecular biology and evolution 24.8 (2007): 1586-1591.

HSDFinder_protocol: Xi Zhang, Yining Hu, David Roy Smith. (2021). HSDFinder: a BLAST-based strategy to search for highly similar duplicated genes in eukaryotic genomes. Frontiers in Bioinformatics. doi: http://doi.org/10.3389/fbinf.2021.803176.

HSDFinder: Xi Zhang, Yining Hu, David Roy Smith. (2021). HSDFinder: a BLAST-based strategy to search for highly similar duplicated genes in eukaryotic genomes. Frontiers in Bioinformatics. doi: http://doi.org/10.3389/fbinf.2021.803176.

HSDecipher: Xi Zhang, Yining Hu, Zhenyu Cheng, John M. Archibald (2023). HSDecipher: A pipeline for comparative genomic analysis of highly similar duplicate genes in eukaryotic genomes. StarProtocols. doi: https://doi.org/10.1016/j.xpro.2022.102014.
